# Biochemical and Molecular Characterization of the Gentisate Transporter GenK in *Corynebacterium glutamicum*

**DOI:** 10.1371/journal.pone.0038701

**Published:** 2012-07-09

**Authors:** Ying Xu, Song-He Wang, Hong-Jun Chao, Shuang-Jiang Liu, Ning-Yi Zhou

**Affiliations:** 1 Key Laboratory of Agricultural and Environmental Microbiology, Wuhan Institute of Virology, Chinese Academy of Sciences, Wuhan, China; 2 State Key Laboratory of Microbial Resources, Institute of Microbiology, Chinese Academy of Sciences, Beijing, China; University of Cambridge, United Kingdom

## Abstract

**Background:**

Gentisate (2,5-dihydroxybenzoate) is a key ring-cleavage substrate involved in various aromatic compounds degradation. *Corynebacterium glutamicum* ATCC13032 is capable of growing on gentisate and *genK* was proposed to encode a transporter involved in this utilization by its disruption in the restriction-deficient mutant RES167. Its biochemical characterization by uptake assay using [^14^C]-labeled gentisate has not been previously reported.

**Methodology/Principal Findings:**

In this study, biochemical characterization of GenK by uptake assays with [^14^C]-labeled substrates demonstrated that it specifically transported gentisate into the cells with *V*_max_ and *K_m_* of 3.06±0.16 nmol/min/mg of dry weight and 10.71±0.11 µM respectively, and no activity was detected for either benzoate or 3-hydoxybenzoate. When GenK was absent in strain RES167 Δ*genK*, it retained 85% of its original transport activity at pH 6.5 compared to that of strain RES167. However, it lost 79% and 88% activity at pH 7.5 and 8.0, respectively. A number of competing substrates, including 3-hydroxybenzoate, benzoate, protocatechuate and catechol, significantly inhibited gentisate uptake by more than 40%. Through site-directed mutagenesis, eight amino acid residues of GenK, Asp-54, Asp-57 and Arg-386 in the hydrophobic transmembrane regions and Arg-103, Trp-309, Asp-312, Arg-313 and Ile-317 in the hydrophilic cytoplasmic loops were shown to be important for gentisate transport. When conserved residues Asp-54 and Asp-57 respectively were changed to glutamate, both mutants retained approximately 50% activity and were able to partially complement the ability of strain RES167 Δ*genK* to grow on gentisate.

**Conclusions/Significance:**

Our results demonstrate that GenK is an active gentisate transporter in *Corynebacterium glutamicum* ATCC13032. The GenK-mediated gentisate transport was also shown to be a limiting step for the gentisate utilization by this strain. This enhances our understanding of gentisate transport in the microbial degradation of aromatic compounds.

## Introduction

Transport of aromatic acids across the microbial cytoplasmic membrane is a step ahead of their catabolism. It is generally accepted that aromatic acids are present in an undissociated form that can diffuse across membranes through passive diffusion under acidic conditions. Most aromatic acids are in their dissociated form under neutral or basic conditions, and thus need to be actively transported across membranes. This procedure is assisted by members of the aromatic acid/H^+^ symporter (AAHS) family within the major facilitator superfamily (MFS) [1]. The AAHS members exhibit 12 transmembrane (TM) α-helices (TM1 to TM12) and conserved motifs are observed in the cytoplasmic loops between transmembrane segments (2–3 and 8–9 loops) [1]. Although the catabolic pathways for a number of aromatic acids have been characterized genetically and biochemically, only three transporters in the AAHS family have so far been functionally identified by uptake assays using their corresponding [^14^C]-labeled substrates: 4-hydroxybenzoate and protocatechuate transporter PcaK from *Pseudomonas putida* PRS2000 [2], benzoate transporter BenK from *Acinetobacter* sp. strain ADP1 [3] and *Corynebacterium glutamicum* [4], and 2,4-dichlorophenoxyacetate transporter TfdK from *Ralstonia eutropha* JMP134 [5]. The important residues for substrate transport in the conserved motifs of PcaK [6], [7] and BenK [4] have also been revealed.

Gentisate (2,5-dihydroxybenzoate) is a typical aromatic acid and also an important ring-cleavage substrate. A number of aromatic compounds have been found to be degraded via the gentisate pathway in phylogenetically divergent strains and the gene clusters encoding the gentisate pathways have also been characterized in several cases. This includes naphthalene metabolism in *Ralstonia* sp. strain U2 [8] and *Polaromonas naphthalenivorans* CJ2 [9], salicylate catabolism in *Ralstonia* sp. strain U2 [10] and *Streptomyces* sp. strain WA46 [11], 3-hydroxybenzoate metabolism in *Rhodococcus* sp. NCIMB12038 [12] and *Corynebacterium glutamicum* [13], and 2,5-xylenol degradation in *Pseudomonas alcaligenes* NCIMB 9867 [14], [15]. Of the identified catabolic gene clusters involved in the gentisate pathway, only one putative gentisate transporter gene (*genK* or formerly as *ncgl2922*) was present in *C. glutamicum*, and the catabolic enzymes involved in the gentisate pathway were found to be induced by gentisate in *C. glutamicum*. [13]. GenK was proposed as a gentisate transporter involved in gentisate assimilation by disruption and complementation in *C. glutamicum* RES167 (a restriction-deficient mutant) [13], and it was also found to be able to confer on *Ralstonia* sp. strain U2 the ability to utilize gentisate [16]. However, its gentisate transport activity has not been identified and characterized biochemically by detection of intracellular [^14^C]-labeled substrate accumulation, a generally recognized practice for functional identification of transporters. In this study, we report the identification of GenK from *C. glutamicum* as a gentisate transporter and its critical residues for gentisate transport activity by uptake assay using [^14^C]-labeled gentisate. This should help to enhance our understanding of gentisate transport in the microbial degradation of aromatic compounds.

## Materials and Methods

### Strains, Plasmids, Media, Growth Conditions and Chemicals

The bacterial strains and plasmids used in this study are listed in Table 1. Aromatic compounds were obtained from Sigma-Aldrich Co. (St. Louis, MO, USA). The tracers, [carboxyl-^14^C] gentisate (55 mCi/mmol), [carboxyl-^14^C] 3-hydroxybenzoate (55 mCi/mmol) and [ring-UL-^14^C] benzoate (70 mCi/mmol) were purchased from American Radiolabeled Chemicals, Inc (St. Louis, MO. USA). Enzymes were purchased from TAKARA Biotechnology Co. Ltd. (Dalian, China). The plasmid DNA extraction kit and DNA gel extraction kit were purchased from OMEGA BIO-TEK Inc. (Doraville, GA. USA). *Escherichia coli* strains were grown in lysogeny broth (LB) at 37°C. *C*. *glutamicum* RES167 and its variants were grown in LB or mineral salts medium (MM) [17], pH 8.4, with 2 mM gentisate or 10 mM glucose, supplemented with 0.05 g L^−1^ of yeast extract to meet the requirement of vitamins for the strains, on a rotary shaker (150 rpm) at 30°C. When necessary, antibiotics were added as follows: nalidixic acid (50 µg/ml for *C. glutamicum*), and chloramphenicol (20 µg/ml for *E*. *coli* and 10 µg/ml for *C. glutamicum*). Isopropyl β-D-1-thiogalactopyranoside (IPTG) and gentisate were used at final concentrations of 1 mM and 0.5 mM, respectively, as inducers.

**Table 1:** Bacterial strains and plasmids used in this study.

Strains or plasmids	Relevant characteristics	Reference or source
Strains		
*C. glutamicum* RES167	Restriction-deficient mutant of *C. glutamicum* ATCC13032, Δ (*cglIM*-*cglIR*-*cglIIR*)	[20]
*C. glutamicum* RES167 Δ*genK*	Derivative of *C. glutamicum* RES167 with DNA fragment encoding for amino acids114–302 of GenK (formerly NCgl2922) deleted	[13]
*C. glutamicum* RES167 Δ*genK*[pXMJ19-*genK*]	*C. glutamicum* RES167 Δ*genK* was complemented with *genK* (formerly *ncgl2922*)	[13]
*E. coli* DH5α	*supE*44 Δ*lacY*169 (ϕ80 *lacZ* ΔM15) *hsdR*17 *recA*1 *endA*1 *gyrA*96 *thi-*1 *relA*1	Gibco, BRL
Plasmids		
pXMJ19	*E. coli*-*C. glutamicum* shuttle vector (Cam^r^ *Ptac lac* ^q^ pBL1 *oriV_C.g_*_._ pK18 *oriV_E.c_*_._)	[19]
pXMJ19-*genK*	PCR fragment containing *genK* (formerly *ncgl2922*) insert into pXMJ19	[13]
pZWXYCg01	PCR fragment containing mutated *genK* (D54A) insert into pXMJ19	This study
pZWXYCg02	PCR fragment containing mutated *genK* (D54E) insert into pXMJ19	This study
pZWXYCg03	PCR fragment containing mutated *genK* (D57A) insert into pXMJ19	This study
pZWXYCg04	PCR fragment containing mutated *genK* (D57E) insert into pXMJ19	This study
pZWXYCg05	PCR fragment containing mutated *genK* (R103A) insert into pXMJ19	This study
pZWXYCg06	PCR fragment containing mutated *genK* (W309V) insert into pXMJ19	This study
pZWXYCg07	PCR fragment containing mutated *genK* (D312A) insert into pXMJ19	This study
pZWXYCg08	PCR fragment containing mutated *genK* (R313A) insert into pXMJ19	This study
pZWXYCg09	PCR fragment containing mutated *genK* (I317H) insert into pXMJ19	This study
pZWXYCg10	PCR fragment containing mutated *genK* (I317Y) insert into pXMJ19	This study
pZWXYCg11	PCR fragment containing mutated *genK* (R386A)insert into pXMJ19	This study

### General Molecular Biology Methods

Plasmid DNA extraction, DNA fragment purification, digestion with restriction endonucleases and ligation with T4 DNA ligase were all conducted in accordance with the manufacturer’s instructions. *E. coli* strains were transformed according to standard procedures [18]. All inserts were sequenced by Invitrogen Biotechnology Co. Ltd (Shanghai, China). Variants of *E*. *coli*-*C*. *glutamicum* shuttle expression vector pXMJ19 [19] were used to electroporate *C. glutamicum* as previously described [20].

### Site-directed Mutagenesis

The desired mutants of GenK were obtained by overlap extension PCR [21]. The outer amplification primers were genK (F): GATTCTAGA **AAAGGAGGACAACC**ATGACATCACACGCACCAG (the XbaI site is underlined and the ribosome binding site is in boldface) and genK (R): ACAGAATTCGCGGAGTTCATCAGAAT (the EcoRI site is underlined). The inner primers were designed to incorporate one codon change. The overlap extension PCR fragments were digested before ligating into the similarly digested pXMJ19. All constructs containing mutated *genK* were sequenced to verify that only the desired mutations had occurred.

### Uptake Assays

The uptake assays of aromatic acids were performed with [^14^C]-labeled substrates as described previously [2] with minor modifications. LB-grown cells of *C. glutamicum* RES167 and its variants were harvested during the exponential phase by centrifugation, after induction with gentisate or IPTG. Cells were resuspended in 50 mM Tris-HCl buffer (pH 8.0) to an optical density at 600 nm (OD_600_) of 5–10 and kept on ice after being washed twice. Before the uptake assay, cells were incubated for 3 min at 30°C with 10 mM glucose for energy generation [22]. The assays were initiated by the addition of 400 µl cell suspension to 600 µl Tris-HCl buffer (50 mM, pH 8.0) containing 40 µM [^14^C]-labeled substrates. Samples (100 µl) were taken at timed intervals and filtered through nucleopore polycarbonate membranes (0.22 µm pore size; Xinya, Shanghai, China), previously soaked with the same buffer containing 400 µM unlabeled substrates, then immediately washed with 2 ml of cold 0.1 M LiCl. For uptake assays performed at different pH, cells were assayed in 50 mM phosphate buffer (pH 6.5, 7.5 and 8.0, respectively). The amount of substrate accumulated in the cells on the filters was determined in a scintillation counter (1450 MicroBeta TriLux, PerkinElmer Life Sciences, Boston, MA, USA). All assays were performed at least in triplicate. In competition and inhibition experiments, uptake measurements were performed in the presence of saturated substrate concentrations (40 µM gentisate) and other possible substrates in 20-fold excess. Apparent *K_m_* and *V*_max_ values were obtained by measuring the uptake of [^14^C]-labeled gentisate in triplicate at 1 min with ten substrate concentrations ranging from 6 to 15 µM. Data were fitted with the Michaelis-Menten equation using the least-squares method [23]. The uptake activity was expressed as nanomole of substrate taken up per milligram of cell dry weight. The biomass concentration was calculated from the OD_600_ values and the correlation factor was taken as 0.25 g cells (dry weight) L^−1^ for an OD_600_ of 1 [24].

### Preparation of Cell Extract and Enzyme Assay

*C. glutamicum* cells were lysed by ultrasonic treatment and the extracts prepared as previously described [8], and the enzyme assay was performed in 50 mM phosphate buffer (pH 7.4) at room temperature. Gentisate 1,2-dioxygenase activity was assayed according to a previous study by measuring the increase in absorbance at 330 nm due to conversion of gentisate to maleylpyruvate [8], the molar extinction coefficient of which was taken as 13,000 M^−1^ cm^−1^ [25]. Protein concentrations were determined by the Bradford method [26] with bovine serum albumin as the standard. One unit of enzyme activity was defined as the amount required for the production of 1 µmol of maleylpyruvate per min at room temperature. Specific activities were expressed as units per milligram of protein.

## Results

### GenK Actively Transports Gentisate

**Figure 1:**
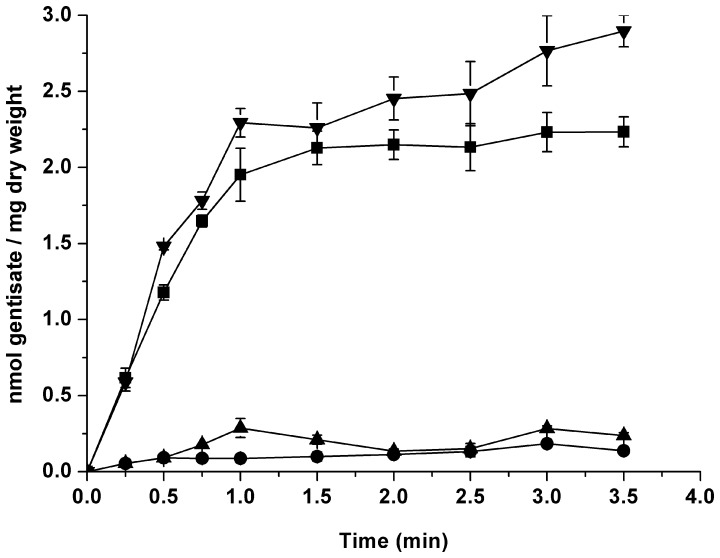
Accumulation of [carboxyl-^14^C] gentisate by *C. glutamicum* RES167 and its variants. *C. glutamicum* RES167 and its variants were grown in LB with gentisate and IPTG induction at 30°C. ▾, *C. glutamicum* RES167 Δ*genK* [pXMJ19-*genK*]; ▪, *C. glutamicum* RES167; •, *C. glutamicum* RES167 Δ*genK*; ▴, *C. glutamicum* RES167 Δ*genK* [pXMJ19]. All points represent the mean value of triplicate trials with error bars denoting the standard deviation.

To detect the capability of transporting gentisate by GenK, intracellular [^14^C]-labeled substrate accumulation through gentisate transport was measured for the resting cells of *C. glutamicum* RES167 and its variants, which were grown in LB with gentisate and IPTG induction. As shown in Figure 1, it was evident that strain RES167 was able to transport [^14^C]-labeled gentisate. However, strain RES167 Δ*genK* (with truncated *genK*) virtually lost the ability to transport gentisate. The transport ability was restored in the complemented strain RES167 Δ*genK* [pXMJ19-*genK*]. The results of the kinetics experiments with [^14^C]-labeled gentisate indicated that strain RES167 transported [^14^C]-labeled gentisate with a *V*_max_ of 3.06±0.16 nmol/min/mg of dry weight, and a *K_m_* of 10.71±0.11 µM. Accumulation detections were also performed with [^14^C]-labeled 3-hydroxybenzoate and benzoate using the above system, but no accumulation of these two aromatic acids by GenK were detected. The *ncgl2920*-encoded gentisate 1,2-dioxygenase catalyzes the initial reaction of gentisate catabolism in *C. glutamicum* [13] and its activity was also assayed from strain RES167 Δ*genK*. It turned out that this enzyme activity was not detected in strain RES167 Δ*genK* but this could be restored by *genK* complementation. The cell extract of the complemented strain RES167 Δ*genK* [pXMJ19-*genK*] exhibited a similar gentisate 1,2-dioxygenase activity of 0.124 U/mg protein to that of the wild-type strain RES167 (0.143 U/mg protein). This suggests that the expression of the gentisate 1,2-dioxygenase gene in *C. glutamicum* was induced by gentisate and this induction was *genK*-dependent.

To investigate the effect of environmental pH values on GenK’s transport activity, uptake assays of [^14^C]-labeled gentisate were also detemined in the first minute at different pH values by LB-grown strain RES167 and its variants with gentisate and IPTG induction. At pH 6.5, the transport activity of gentisate by strain RES167 Δ*genK* still retained 85%, in comparison with that of strain RES167 (1.87±0.18 nmol/min/mg of dry weight). However, transport activities of strain RES167 Δ*genK* were almost suppressed at pH 7.5 and 8.0, with losses of 79% and 88% activity, respectively, in comparison with those of strain RES167 (2.49±0.30 and 2.57±0.13 nmol/min/mg of dry weight, respectively, at pH 7.5 and pH 8.0). Notably, strain RES167 Δ*genK* [pXMJ19-*genK*] had a slightly higher rate for transporting gentisate (2.07±0.16, 2.58±0.20 and 2.72±0.12 nmol/min/mg of dry weight at pH 6.5, 7.5 and 8.0, respectively) than strain RES167.

### Identification of the Critical Residues in GenK for Gentisate Transport

**Figure 2:**
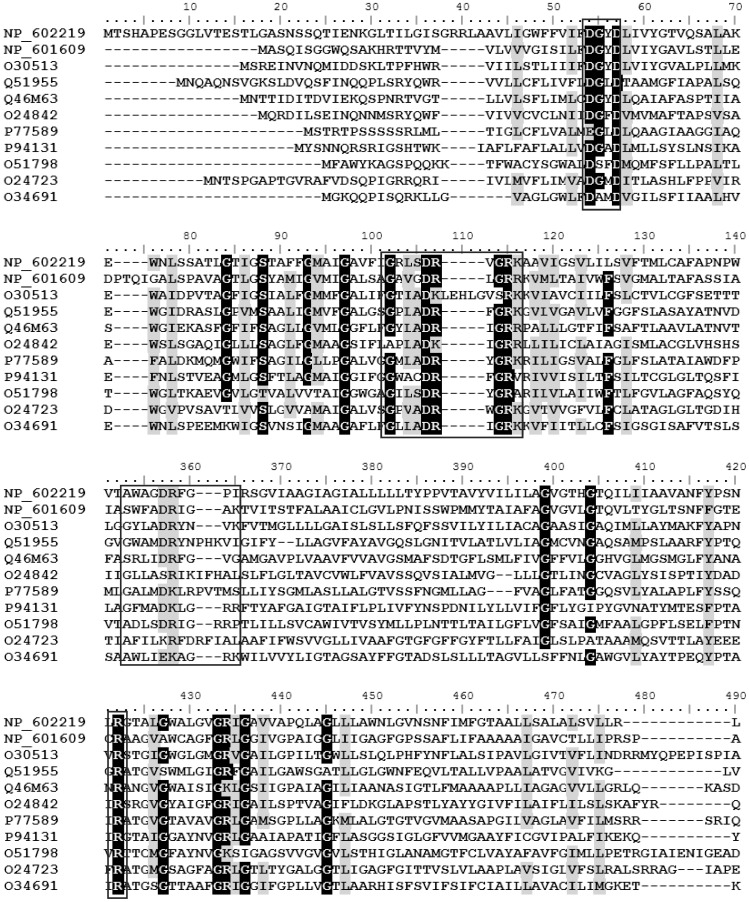
Sequence alignments of the AAHS family members by Clustal W. The consensus profiles identified are highlighted. The black-marked residues indicate identical amino acid residues and the gray-marked residues indicate similar amino acid residues with a similarity threshold of 75%. The residues for site-directed mutagenesis are in the motifs which are surrounded in the rectangle. The accession numbers of all the AAHS family members in the TCDB database (http://www.tcdb.org/) for comparison are as follows: GenK (accession no. NP_602219), BenK (accession no. NP_602219 and O30513), PcaK (accession no. Q51955), TfdK (accession no. Q46M63), VanK (accession no. O24842), MhpT (accession no. P77589), MucK (accession no. P94131), MmlH (accession no. O51798), Orf1 (accession no. O24723), YceI (accession no. O34691).

**Figure 3:**
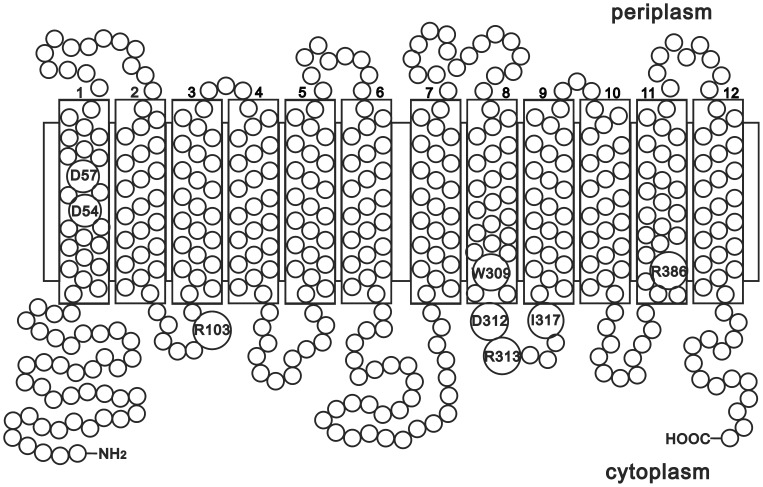
Transmembrane segment prediction for GenK based on the result of bioinformatic analysis by TMHMM. Predicted hydrophobic transmembrane segments are enclosed in boxes. The amino acid residues of GenK for site-directed mutagenesis in this study are marked.

**Figure 4:**
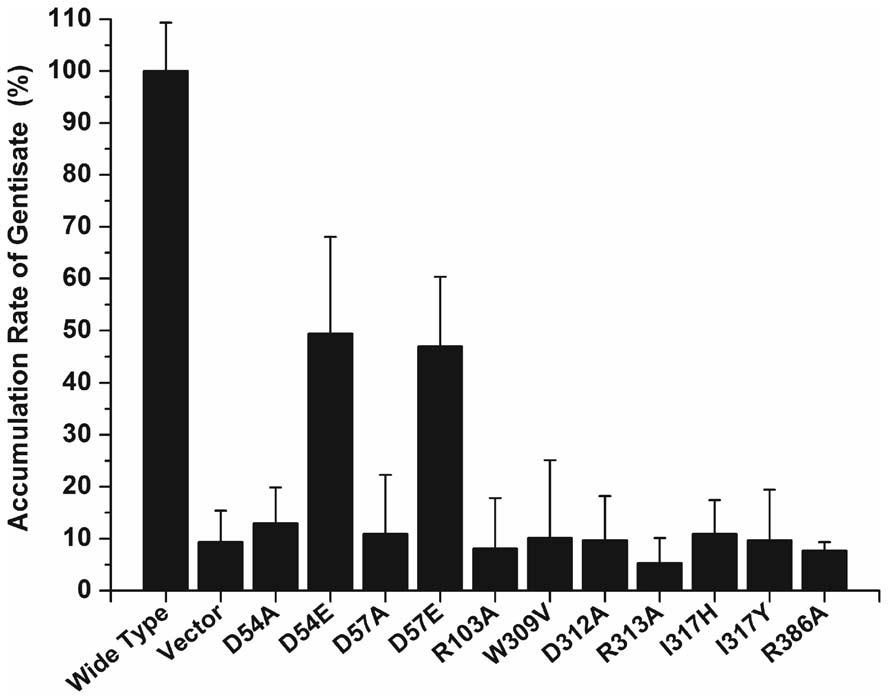
Accumulation rate of gentisate by *C. glutamicum* RES167Δ*genK* expressing wild-type and mutant GenK proteins. Proteins were expressed in *C. glutamicum* RES167 Δ*genK* cells from the *E. coli*-*C. glutamicum* shuttle vector pXMJ19 constructs. All strains were grown in LB with gentisate and IPTG induction. The rate of accumulation of gentisate by strain RES167 Δ*genK* [pXMJ19-*genK*] containing wild-type GenK protein (2.47±0.23 nmol/min/mg of dry weight) was set as 100%. Wild Type, wild-type GenK in strain RES167 Δ*genK* [pXMJ19-*genK*]; D54E, mutant GenK of Asp-54 substituted with glutamate in strain RES167 Δ*genK* [pZWXYCg02]; D57E, mutant GenK of Asp-57 substituted with glutamate in strain RES167 Δ*genK* [pZWXYCg04]. All assays were performed in triplicate and standard deviations are represented by error bars.

Alignment of all the AAHS family members in the TCDB database (http://www.tcdb.org/) by Clustal W (Figure 2) showed that there were a number of conserved amino acid residues. The topology prediction by TMHMM (http://www.cbs.dtu.dk/services/TMHMM/) indicated that all AAHS family members contained 12 α-helix transmembrane spanners. The conserved motifs in the cytoplasmic hydrophilic loops and the transmembrane hydrophobic regions were known to be important for substrate transport [4], [6], [7]. In order to identify the critical residues of GenK for gentisate transport, a series of site-directed mutants were generated in two cytoplasmic hydrophilic loops (2–3 and 8–9 loops) and the transmembrane hydrophobic regions of GenK as marked in Figure 3. The arginine (Arg-103) within the 2–3 loop, was changed to an alanine (R103A). In the 8–9 loop, Trp-309 was substituted with a valine (W309V), Asp-312 and Arg-313 were both changed to alanine residues (D312A and R313A), and Ile-317 was substituted with histidine and tyrosine (I317H and I317Y), respectively. Asp-54 and Asp-57 in the DGXD motif of TM1 were both substituted with alanine residues (D54A and D57A) and glutamate residues (D54E and D57E), respectively, and Arg-386 in TM11 was changed to an alanine residue (R386A). Each mutant *genK* was expressed in *C. glutamicum* RES167 Δ*genK* using the vector pXMJ19. As shown in Figure 4, the uptake assays in the first minute showed that all mutants were devoid of gentisate transport activity except mutants D54E and D57E which retained approximately 50% activity in comparison with that of wild-type GenK.

### GenK Mutants D54E and D57E Partially Complemented the Ability of Strain RES167 Δ*genK* to Grow on Gentisate

**Figure 5:**
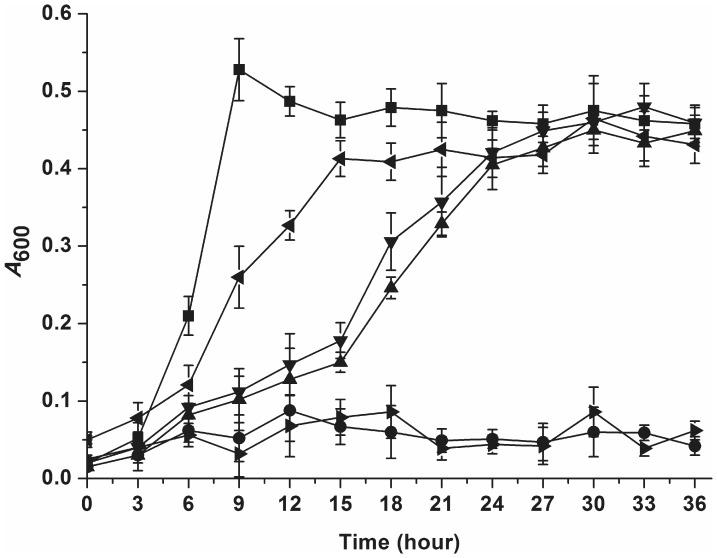
Growth curves of *C*. *glutamicum* strains containing mutant GenK D54E and D57E. All strains were grown with 2 mM gentisate in MM (pH 8.4) at 30°C: ▪, *C. glutamicum* RES167; ◂, *C. glutamicum* RES167 Δ*genK* [pXMJ19-*genK*]; ▴, *C. glutamicum* RES167 Δ*genK* [pZWXYCg02]; ▾, *C. glutamicum* RES167 Δ*genK* [pZWXYCg04]; •, *C. glutamicum* RES167 Δ*genK*; ▸, *C. glutamicum* RES167 Δ*genK* [pXMJ19]. All points represent the mean value of triplicate trials with error bars denote standard deviation.

Given the fact that the two mutants D54E and D57E retained 50% of the original transport activity, it was interesting to determine if they were able to complement the ability of *genK*-truncated strain RES167 Δ*genK* to grow on gentisate. The complemented strains were grown with 2 mM gentisate in MM (pH 8.4) at 30°C. The results indicated that strains RES167 Δ*genK* [pZWXYCg02] (containing D54E) and RES167 Δ*genK* [pZWXYCg04] (containing D57E) were able to grow on gentisate, although both had lower growth rates than those of strain RES167 Δ*genK* [pXMJ19-*genK*] (containing wild-type GenK) and the wild-type strain as shown in Figure 5. It took 27 hours for these strains containing mutant GenK to reach an OD_600_ of 0.4 when grown on gentisate, in contrast to 15 and 9 hours respectively for strain RES167 Δ*genK* [pXMJ19-*genK*] and the wild-type strain.

### Competing Substrates Inhibited Gentisate Uptake by GenK

**Figure 6:**
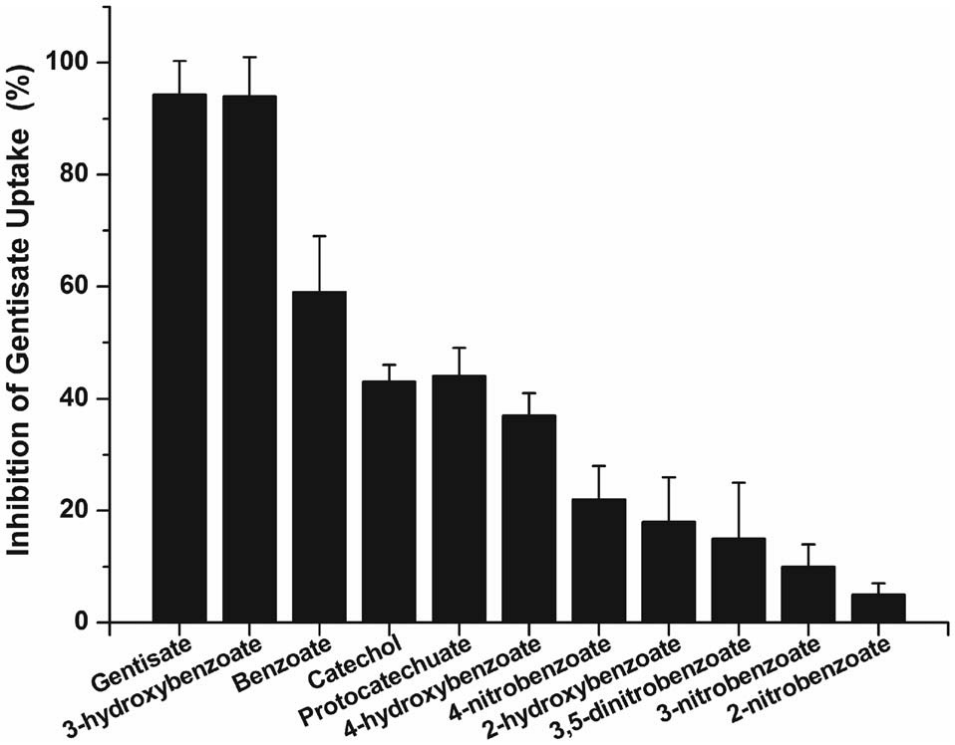
Substrate inhibition of GenK-mediated gentisate uptake in *C. glutamicum* RES167. The concentrations of gentisate and competing substrate were 40 µM and 800 µM, respectively. The rate of accumulation of gentisate (without competing substrates) was 2.19±0.14 nmol/min/mg of cell dry weight, which was set as 100%. Inhibition was determined by comparing the rate of gentisate uptake in the absence and presence of competing substrate. Values are the averages from three experiments and standard deviations are represented by error bars.

In order to detect whether the gentisate transport by GenK was inhibited by other aromatic compounds, uptake assays with [^14^C]-labeled gentisate by *C. glutamicum* RES167 were performed in the presence of a 20-fold excess of gentisate structural analogues (Figure 6). In a control experiment, unlabeled gentisate inhibited [^14^C]-labeled gentisate uptake activity by 94.3%. 3-Hydroxybenzoate inhibited gentisate transport activity by 94%. Benzoate, catechol and protocatechuate inhibited about 40–60% of the uptake activity. Other compounds tested had less inhibition effects on the uptake activity as shown in Figure 6.

## Discussion

In comparison to the characterized microbial catabolic pathways of aromatic acids, studies on their transport have received considerably less attention. The gentisate transporter GenK is the fourth identified aromatic acid transporter, in addition to 4-hydroxybenzoate and protocatechuate transporter PcaK [2], benzoate transporter BenK [3], [4] and 2,4-dichlorophenoxyacetate transporter TfdK [5]. Although GenK was tentatively proposed as a gentisate transporter based on experiments where its encoding gene was disrupted and complemented in *C. glutamicum* RES167 [13], its biochemical characterization by uptake assay using [^14^C]-labeled gentisate was not reported until this study.

Bioinformatic analysis showed that GenK contains 12 typical transmembrane hydrophobic α-helical regions (TM1 to TM12). This characteristic feature of MFS members is important for packing to form the perimeter of a pore through which the substrate crosses the cell membrane [27], [28], [29]. It has previously been proven that the conserved motifs in the cytoplasmic hydrophilic loops and the transmembrane hydrophobic regions were known to be important for substrate transport [4], [6], [7]. Site-directed mutagenesis at conserved residues should make conformational changes in the transporters, affecting the substrate translocation across the membrane [30]. In the cytoplasmic hydrophilic loops (between the transmembrane hydrophobic α-helical regions), the negative charged aspartate (Asp-312 of GenK and BenK, Asp-323 of PcaK) in the 8–9 loop was conserved in the corresponding site among the entire AAHS family. It is not surprising that the mutation of this residue to a simple and uncharged alanine in GenK resulted in a complete loss of its transport activity, similar to the cases for PcaK [6] and BenK [4]. However, mutants of the positive charged Arg-103 (in 2–3 loop) and Arg-313 (in 8–9 loop) to the uncharged aliphatic amino acid alanine in GenK, which are the other two strictly conserved residues in the AAHS family but with no previous functional studies, were also devoid of transport activity. This suggested that these two residues may also play important roles in gentisate transport by GenK, and probably it is also the case for other AAHS members. In the partially conserved motif of the 8–9 loop of GenK, two non-conserved residues, the aromatic amino acid Trp-309 and the aliphatic amino acid Ile-317, were also suggested to be essential for gentisate transport when the activity was lost after the uncharged aromatic amino acid Trp-309 was changed to an uncharged aliphatic amino acid valine, and the uncharged aliphatic amino acid Ile-317 was changed to a charged heterocyclic amino acid histidine or an uncharged aromatic amino acid tyrosine. The significant changes in amino acids properties at these two sites of GenK may have resulted in the loss of its gentisate transport activity. Although the characteristic features in the 8–9 loop were partially conserved in this family [6], the mutations in these non-conserved residues may have impacted on the specific biding between the substrate gentisate and its transporter GenK in this case. It would be interesting to determine whether the mutations of the corresponding residues in other AAHS members also have the same effect on their transport activity as that of GenK. All of the above mutations caused huge changes in the amino acid characteristics. These may cause tertiary structural changes in GenK and result in the prevention of gentisate from crossing the cell membrane.

In the transmembrane hydrophobic regions of GenK, when the two conserved charged residues Asp-54 and Asp-57 were replaced by uncharged alanines, the gentisate transport activity was virtually lost. When the above two aspartate residues were replaced by glutamate, however, approximately 50% of the activity was still retained. These observations were similar to those in the previous study of 4-hydroxybenzoate and protocatechuate transport by PcaK [7]. This may be owing to the fact that aspartate and glutamate are both negatively charged amino acids with a similar R-group, and substitution with similar residues may reduce the impact on its activity. The GenK mutants D54E and D57E were re-introduced to strain RES167 Δ*genK* to investigate whether they would support the *genK*-truncated strain to grow on gentisate, which was not performed for the equivalent PcaK mutants [7]. The strain containing either D54E or D57E was still able to grow on gentisate but with a decreased growth rate, in comparison with the strain RES167 Δ*genK* [pXMJ19-*genK*] (containing wild-type GenK), suggesting that the substrate transport is likely a limiting step for the gentisate utilization by this strain.

Since aromatic acids can diffuse across biological membranes, active transport is perhaps theoretically unnecessary [31]. As a typical aromatic acid with two hydroxyl groups, the pKa of gentisate is 3.194 (ACD/Labs Software). Therefore, gentisate is in the undissociated form that should be able to diffuse across membranes through passive diffusion under acidic conditions. However, under neutral or alkaline conditions, less than 0.1% gentisate is in the undissociated form, and active transport is necessary. In this study, the dramatic decrease in the transport activity of strain RES167 Δ*genK* has also been observed when the pH value was increased from 6.5 to 8.0. This suggested that the environmental pH may be one of the decisive factors for the requirement of a transporter during the degradation of an aromatic acid. Despite the fact that the gentisate pathway appears in the degradation of many aromatic compounds of different bacteria genera [8], [11], [12], [13], gentisate transport has to date only been found in *C. glutamicum*. The presence of a particular transporter in a strain may increase the efficiency of substrate uptake, and may have a growth advantage in natural environments where this compound exists at a low concentration [2]. The presence of *genK*, in addition to the gentisate catabolism-encoding genes, has enabled *C. glutamicum* ATCC13032 to grow on gentisate. In contrast, despite the presence of all the necessary genes encoding for gentisate catabolism, *Ralstonia* sp. strain U2 [16] and *Streptomyces* sp. strain WA46 [11] are not able to grow on gentisate, apparently caused by the lack of a gentisate transporter.
